# Deletion of Smad3 improves cardiac allograft rejection in mice

**DOI:** 10.18632/oncotarget.4849

**Published:** 2015-07-13

**Authors:** Ying-ying Wang, Hong Jiang, Yu-cheng Wang, Xiao-ru Huang, Jun Pan, Chen Yang, Zhang-fei Shou, Shi-long Xiang, Da-jin Chen, Hui-yao Lan, Jiang-hua Chen

**Affiliations:** ^1^ Kidney Disease Center, The First Affiliated Hospital, School of Medicine, Zhejiang University, Hangzhou, China; ^2^ Li Ka Shing Institute of Health Sciences, and Department of Medicine and Therapeutics, The Chinese University of Hong Kong, Hong Kong, China; ^3^ Shenzhen Research Institute, The Chinese University of Hong Kong, Shenzhen, China; ^4^ Key Laboratory of Combined Multi-organ Transplantation, Ministry of Public Health, State Key Laboratory for Diagnosis and Treatment of Infectious Diseases, Division of Hepatobiliary and Pancreatic Surgery, The First Affiliated Hospital, School of Medicine, Zhejiang University, Hangzhou, China

**Keywords:** Smad3, cardiac allograft rejection, Th1, Th2, Th17, Pathology

## Abstract

T cells play a critical role in acute allograft rejection. TGF-β/Smad3 signaling is a key pathway in regulating T cell development. We report here that Smad3 is a key transcriptional factor of TGF-β signaling that differentially regulates T cell immune responses in a mouse model of cardiac allograft rejection in which donor hearts from BALB/c mice were transplanted into Smad3 knockout (KO) and wild type (WT) mice. Results showed that the cardiac allograft survival was prolonged in Smad3 KO recipients. This allograft protection was associated with a significant inhibition of proinflammatory cytokines (IL-1β, TNF-α, and MCP-1) and infiltration of neutrophils, CD3^+^ T cells, and F4/80^+^ macrophages. Importantly, deletion of Smad3 markedly suppressed T-bet and IFN-γ while enhancing GATA3 and IL-4 expression, resulting in a shift from the Th1 to Th2 immune responses. Furthermore, mice lacking Smad3 were also protected from the Th17-mediated cardiac injury, although the regulatory T cell (Treg) response was also suppressed. In conclusion, Smad3 is an immune regulator in T cell-mediated cardiac allograft rejection. Loss of Smad3 results in a shift from Th1 to Th2 but suppressing Th17 immune responses. Thus, modulation of TGF-β/Smad3 signaling may be a novel therapy for acute allograft rejection.

## INTRODUCTION

Organ transplantation is the final definitive treatment for the end stage of many organ diseases. However, allograft rejection remains the main impediment in organ transplantation clinically. As immune regulatory cells, CD4^+^ T helper (Th) cells play a critical role in transplant rejection, especially in acute allograft rejection [1]. This is supported by the finding that adoptive transfer of CD4^+^ T cells promotes but depletion of this T cell population inhibits allograft rejection [2, 3]. It is now clear that naive CD4^+^ T cells can proliferate and differentiate into Th cells including Th1, Th2, T regulatory (Treg) cells, and interleukin 17 (IL-17)-producing Th cells (Th17). Recent studies indicated that transforming growth factor-β (TGF-β) is a master regulator in T cell development, homeostasis, tolerance, and differentiation during the immune response [4]. In mammals, 3 members of the TGF-β family (TGF-β1, TGF-β2, and TGF-β3) have been identified, with TGF-β1 being the predominant form expressed in the immune system [5]. Binding of TGF-β1 to its receptor II (TβRII) can activate the TGF-β receptor type I (TβRI)-kinase, resulting in the phosphorylation of Smad2 and Smad3, two receptor-associated Smads. Subsequently, phosphorylated Smad2 and Smad3 bind to the Smad4 and form the Smad complex, which translocates into the nucleus to exhibit its diverse biological activities under disease conditions [6]. Among Smad signals, Smad3 is a key regulator in CD4^+^ T cell differentiation as deletion of Smad3 impairs T cell immunity [7]. However, the function of Smad3 in regulating immune response during organ transplantation rejection remains largely unknown. In the present study, we tested the hypothesis that Smad3 may be a key regulator of TGF-β signaling in cardiac transplantation rejection. The hypothesis was examined in an acute cardiac allograft rejection induced in Smad3 KO mice and the regulatory mechanisms of Smad3 in acute cardiac rejection were investigated.

## RESULTS

### Loss of Smad3 inhibits cardiac inflammation and promotes cardiac allograft survival

**Figure 1:**
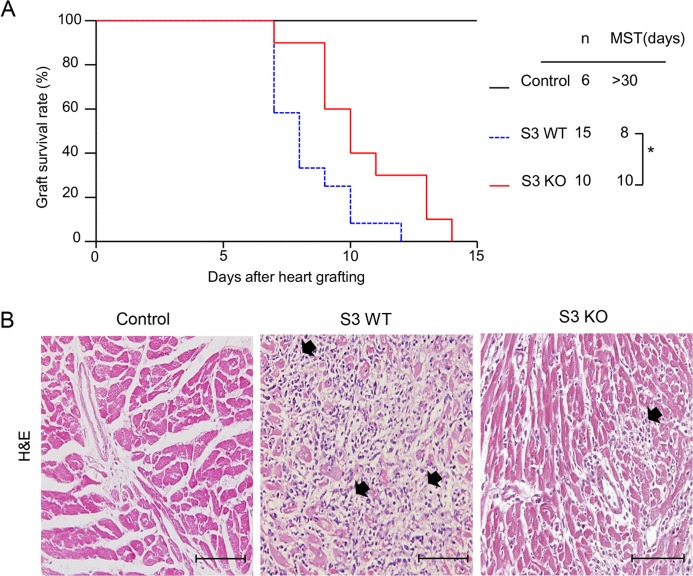
Deletion of Smad3 from the recipients significantly improves cardiac allograft rejection **A.** Cardiac allograft survival rate. **B.** Histologic changes (H&E staining) in cardiac allografts on postoperative days (POD) 7, black arrows show the loci of myocyte damage. Results show that deletion of Smad3 from the recipients results in prolonged allograft survival and less histological damage. *p < 0.05 versus Smad3 WT mice; MST, median survival time; Scale bar = 100 μm.

**Figure 2:**
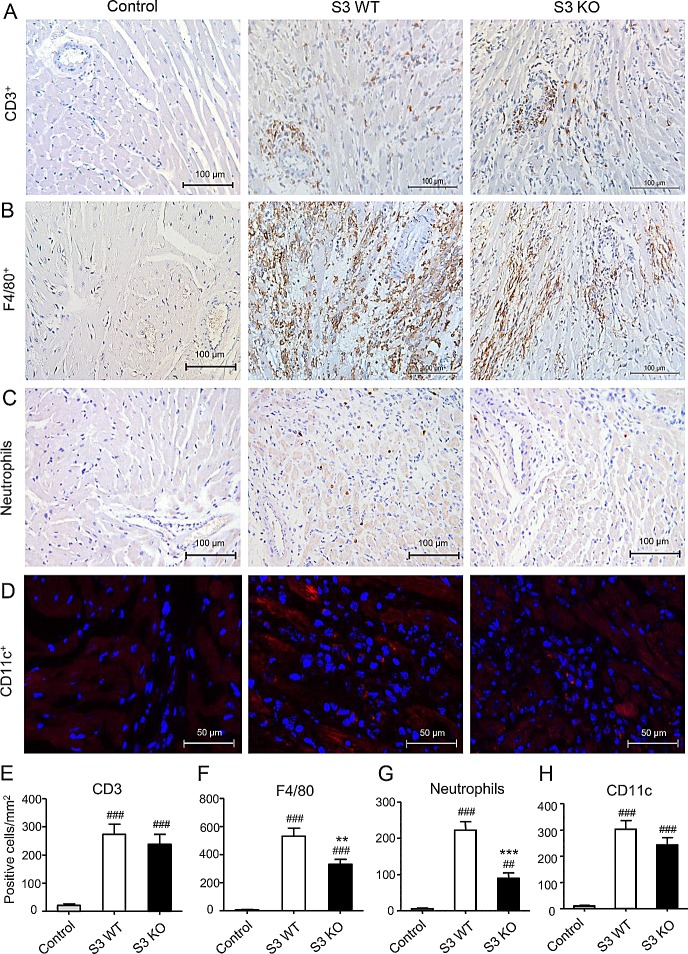
Deletion of Smad3 inhibits infiltration of inflammatory cells in cardiac allograft rejection **A.** Immunohistochemistry shows CD3^+^ T cells, **B.** F4/80^+^ macrophages, **C.** neutrophils, and **D.** CD11c^+^ dendritic cells infiltration in cardiac allografts on POD 7. (E-H) Quantitative data of CD3^+^ cells, F4/80^+^ cells, neutrophils and CD11c^+^ cells infiltrating the allograft tissues. Data represent mean ± SEM for groups of 6 mice. **p < 0.01, ***p < 0.01 versus Smad3 WT mice; ##*p* < 0.001, ###*p* < 0.001 versus control mice (BALB/c mice received cardiac allografts from BALB/c mice).

**Figure 3:**
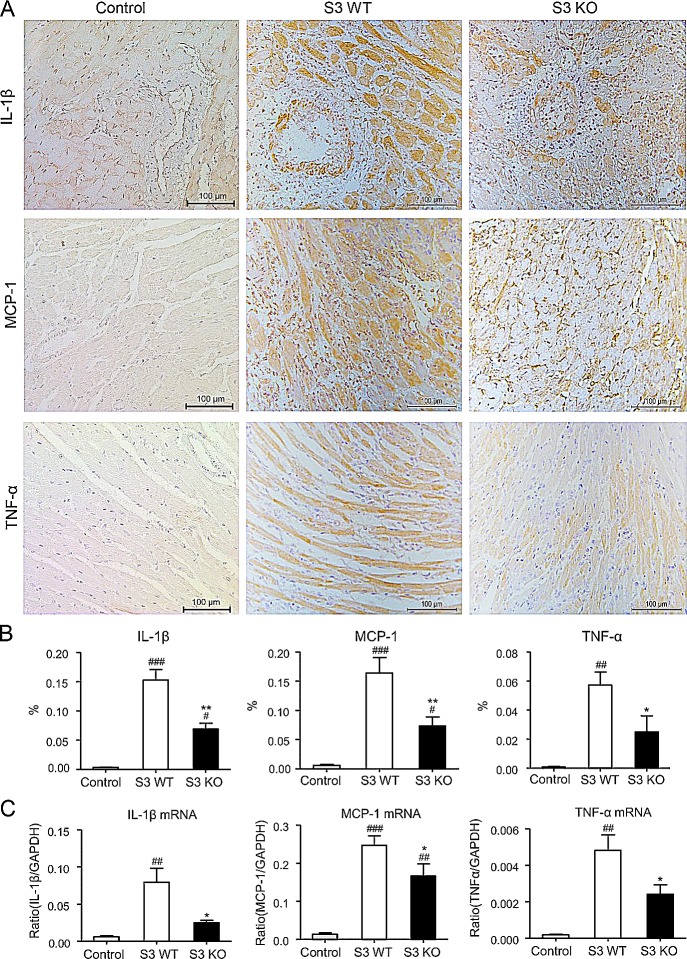
Improvement of cardiac allograft rejection in Smad3 deficient recipients is associated with inhibition of upregulation of proinflammatory cytokines **A.** Expression levels of IL-1β, MCP-1 and TNF-α in cardiac allografts on POD 7 by immunohistochemistry. **B.** Quantitative data of IL-1β, MCP-1 and TNF-α from immunohistochemically stained tissues. **C.** Real-time PCR for IL-1β, MCP-1 and TNF-α mRNA expression in cardiac allografts on POD 7. Data are expressed as the mean ± SEM. *p < 0.05, **p < 0.01 versus Smad3 WT mice; #*p* < 0.05, ##*p* < 0.01, ###*p* < 0.001 versus control mice.

To investigate the role of Smad3 in acute allograft rejection, we transplanted the heart from BALB/c (H-2^d^) mice into Smad3 KO or Smad3 WT (B6 background, H-2^b^) mice, or control BALB/c mice. When recipients were BALB/c mice, the cardiac grafts survived for more than 30 days without detectable cardiac inflammation. However, histology and immunohistochemistry detected that cardiac allografts in Smad3 WT mice developed a severe acute rejection with many F4/80^+^ macrophages and neutrophils infiltration and a marked upregulation of proinflammatory cytokines including IL-1β, TNF-α, and MCP-1, resulting in the lower graft survival rate (Figures 1-3). In contrast, cardiac allografts in Smad3 KO mice showed a less severe allograft rejection with moderate macrophages infiltration, a few neutrophils, and inhibition of IL-1β, TNF-α, and MCP-1 expression, resulting in an improvement of allograft survival (Figures 1-3). Interestingly, mice lacking Smad3 did not alter CD11c^+^ dendritic cells and CD3^+^ T cells infiltrating the graft when compared to the littermate Smad3 WT mice (Figure 2A, 2D, 2E and 2H).

### Deletion of Smad3 results in a shift from Th1 to Th2 immune response in the donor heart with acute cardiac allograft rejection

**Figure 4:**
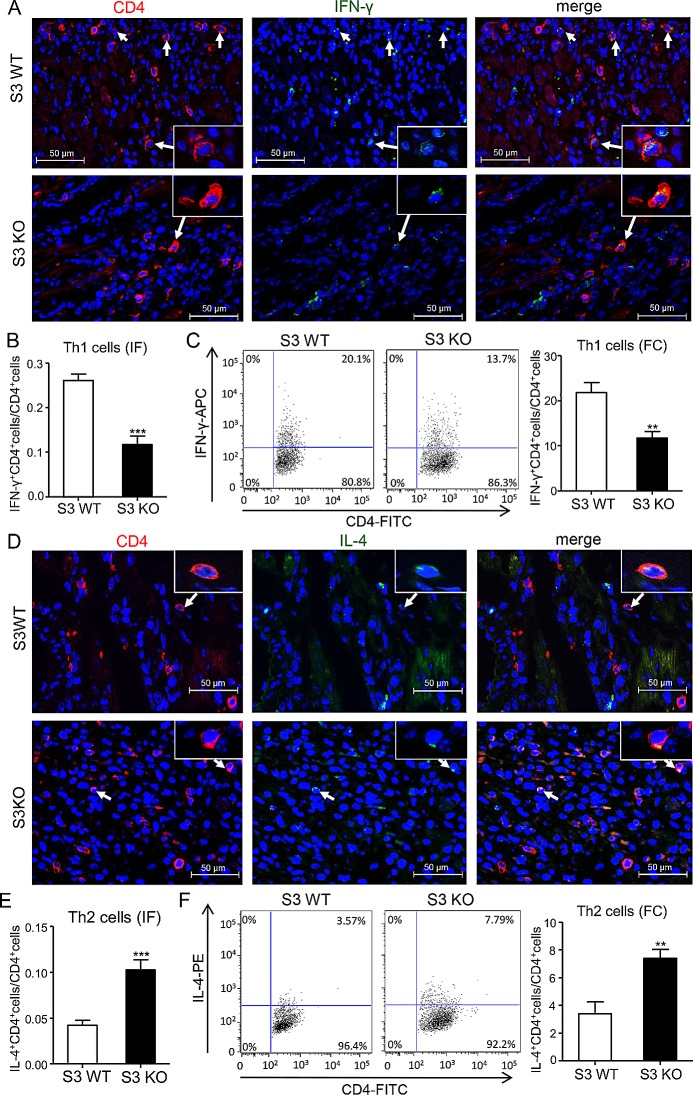
Deletion of Smad3 suppresses Th1 but enhances Th2 cells infiltrating the cardiac allografts **A.** Two-color immunofluorescence for detecting Th1 cells (CD4^+^IFN-γ^+^) infiltrating the allografts on POD 7. **B.** Quantitative data of Th1 cells on two-color immunofluorescence-staining sections. **C.** Quantitative analysis of CD4^+^IFN-γ^+^ cells by two-color flow cytometry in cardiac allografts of Smad3 KO and WT recipients on POD 7. **D.** Two-color immunofluorescence for detecting Th2 cells (CD4^+^IL-4^+^) infiltrating the allografts on POD 7. **B.** Quantitative data of Th2 cells on two-color immunofluorescence-staining sections. **C.** Quantitative analysis of CD4^+^IL-4^+^ cells by two-color flow cytometry in cardiac allografts of Smad3 KO and WT recipients on POD 7. Data are expressed as the mean ± SEM. **p < 0.01, ***p < 0.001 versus Smad3 WT mice.

**Figure 5:**
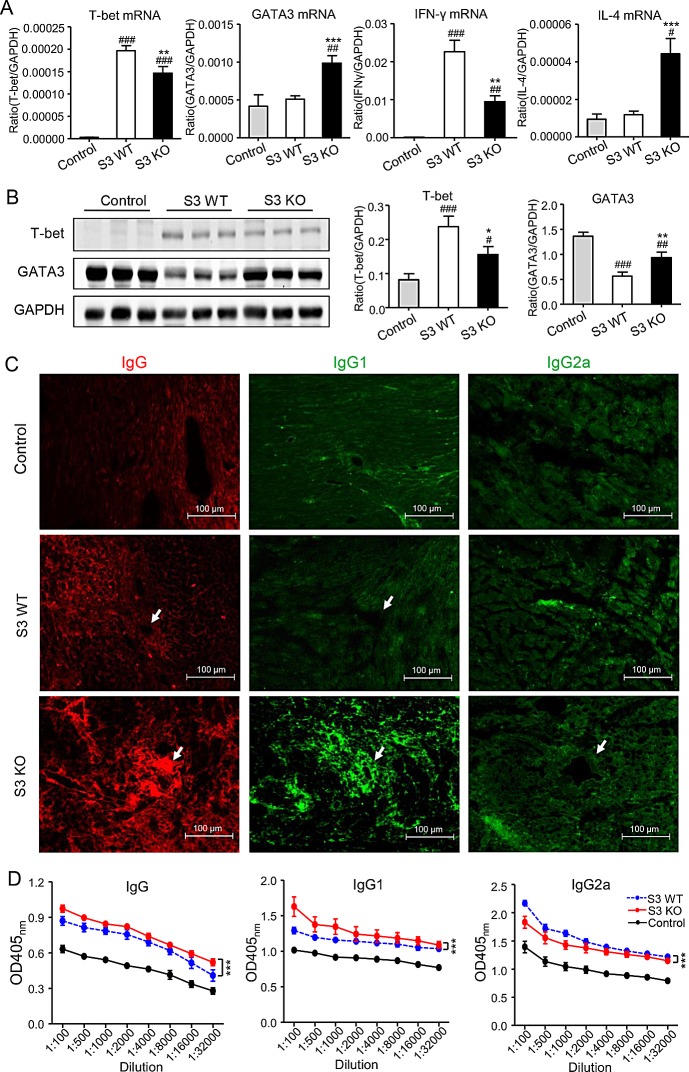
Smad3-deficient mice are more likely to suffer a Th2-type immune response, while suppressing Th1-type immune response in cardiac allografts **A.** Real-time PCR for T-bet, GATA3, IFN-γ and IL-4 mRNA expression in cardiac allografts on POD 7. **B.** Western blot analysis shows the expression of T-bet and GATA3 in cardiac allografts in control, Smad3 KO and WT recipients on POD 7. **C.** Immunofluorescence shows the total IgG, IgG1 and IgG2a deposition within the cardiac allograft tissues on POD 7. **D.** Serum levels of IgG, IgG1 and IgG2a in control, Smad3 KO and WT recipients on POD 7 detected by ELISA. Data are expressed as the mean ± SEM. *p < 0.05, **p < 0.01, ***p < 0.001 versus Smad3 WT mice; #*p* < 0.05, ##*p* < 0.01, ###*p* < 0.001 versus control mice.

We next investigated the immunological mechanisms whereby deletion of Smad3 from the recipients improves cardiac allograft survival. We first examined the Th1 versus Th2 immune response in the grafted donor heart in Smad3 KO or WT mice. Two-color immunofluorescence and flow cytometry analysis found that although the number of CD4^+^ T cells infiltrating the cardiac graft were not significantly different between Smad3 KO and WT mice, mice lacking Smad3 showed a great suppression of the Th1 immunity in the grafted donor heart as demonstrated by a 40% reduction in the number of CD4^+^IFN-γ^+^ cells (Figure 4A-4C). In contrast, Th2 immune response was largely promoted in the grafted donor heart in Smad3 KO mice as evidenced by a 50% increase in the number of CD4^+^IL-4^+^ cells (Figure 4D-4F). Further studies by real-time PCR and western blot analysis also detected a marked suppression of the Th1 master transcriptional factor T-bet and its signature cytokine IFN-γ in the grafted heart of Smad3 KO mice (Figure 5A and 5B), which was associated with a large increase in the Th2 immune response by doubling levels of GATA3 and IL-4 (Figure 5A and 5B). These results suggested that deletion of Smad3 results in a shift of the graft immunity from the Th1 to Th2 during acute allograft rejection. This was further supported by the finding that mice lacking Smad3 promoted Th2-dependent humoral immune response with higher levels of IgG, IgG1, but lower levels of IgG2a deposition within the donor heart (Figure 5C). ELISA supported this notion that mice null for Smad3 developed higher levels of serum IgG and IgG1 but lower level of IgG2a when compared to Smad3 WT (Figure 5D).

**Figure 6:**
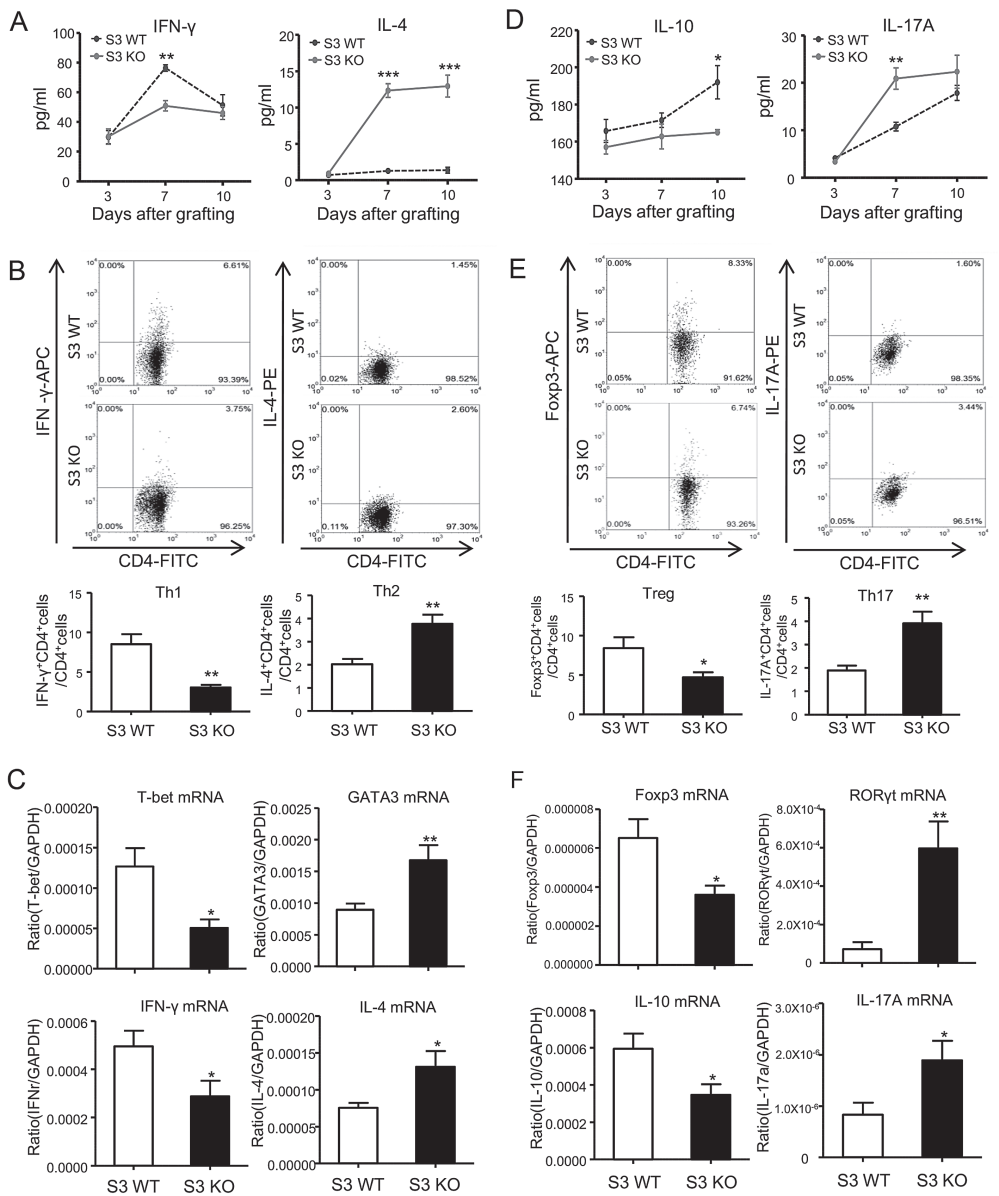
Deletion of Smad3 differentially regulates T cell differentiation during cardiac allograft rejection **A.**, **D.** Serum levels of IFN-γ, IL-4, IL-10 and IL-17A in Smad3 KO and WT recipients on POD 7 detected by ELISA. **B.**, **E.** Quantitative analysis of CD4^+^IFN-γ^+^ cells, CD4^+^IL-4^+^ cells, CD4^+^Foxp3^+^ cells and CD4^+^IL-17A^+^ cells by two-color flow cytometry analysis in the spleen of Smad3 KO and WT recipients on POD 7. **C.**, **F.** Real-time PCR analysis of T-bet, GATA3, Foxp3, RORγt, IFN-γ, IL-4, IL-10 and IL-17A mRNA expression in the spleen of Smad3 KO and WT recipients on POD 7. Data are expressed as the mean ± SEM. *p < 0.05, **p < 0.01, ***p < 0.001 versus Smad3 WT mice.

To address whether the alternation of Th1 and Th2 immune responses within the cardiac allografts of Smasd3 KO recipients is associated with T cell differentiation systemically, we detected the associated cytokines in serum by ELISA, and analyzed Th1 and Th2 responses in the spleen transcriptionally by real-time PCR and phenotypically by two-color flow cytometry analysis. It showed that deletion of Smad3 suppressed the Th1 but promoted the Th2 responses in the spleen during cardiac allograft rejection (Figure 6A-6C).

### Deletion of Smad3 suppresses Th17-mediated acute cardiac allograft rejection, while impairing the Treg response

**Figure 7:**
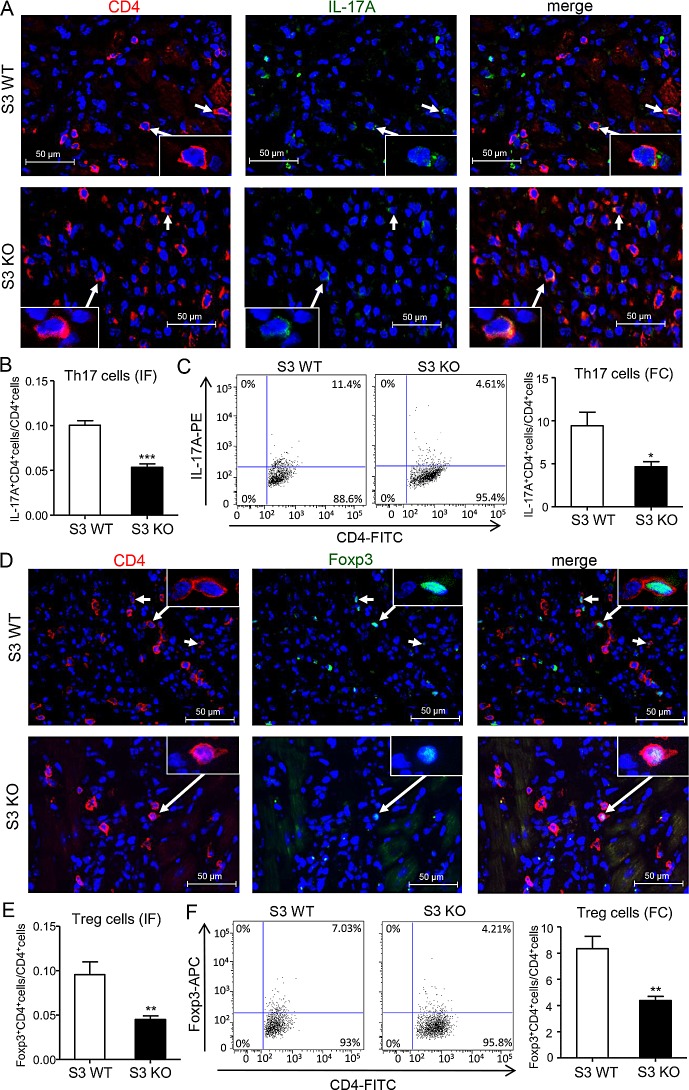
Deletion of Smad3 suppresses Th17 and Treg cells infiltrating the cardiac allografts **A.** Two-color immunofluorescence for detecting Th17 cells (CD4^+^IL17A^+^) infiltrating the allografts on POD 7. **B.** Quantitative data of Th17 cells on two-color immunofluorescence-staining sections. **C.** Quantitative analysis of CD4^+^IL17A^+^ cells by two-color flow cytometry in cardiac allografts of Smad3 KO and WT recipients on POD 7. **D.** Two-color immunofluorescence for detecting Treg cells (CD4^+^Foxp3^+^) infiltrating the allografts on POD 7. **B.** Quantitative data of Treg cells on two-color immunofluorescence-staining sections. **C.** Quantitative analysis of CD4^+^Foxp3^+^ cells by two-color flow cytometry in cardiac allografts of Smad3 KO and WT recipients on POD 7. Data are expressed as the mean ± SEM. *p < 0.05, **p < 0.01, ***p < 0.001 versus Smad3 WT mice.

**Figure 8:**
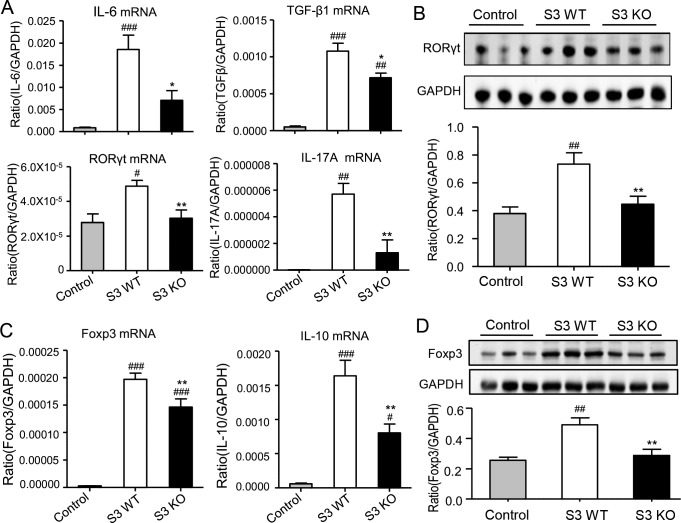
Deletion of Smad3 from the recipients suppresses Th17 and Treg immune response in cardiac allografts **A.** Real-time PCR for IL-6, TGF-β1, RORγt, and IL-17A mRNA expression in cardiac allografts on POD 7. **B.** Western blot analysis shows the expression of RORγt in cardiac allografts in control, Smad3 KO and WT recipients on POD 7. **C.** Real-time PCR for Foxp3 and IL-10 mRNA expression in cardiac allografts on POD 7. **D.** Western blot analysis shows the expression of RORγt in cardiac allografts in control, Smad3 KO and WT recipients on POD 7. Data are expressed as the mean ± SEM. *p < 0.05, **p < 0.01 versus Smad3 WT mice; #*p* < 0.05, ##*p* < 0.01, ###*p* < 0.001 versus control mice.

Since TGF-β1, together with IL-6, plays a critical role in regulating Th17 immune response [8], we thus examined whether Smad3 functions to regulate the Th17-mediated allograft rejection in the donor heart. Two-color immunofluorescence and flow cytometry analysis revealed a strong Th17 immune response with many CD4^+^IL-17A^+^ cells in the donor heart of Smad3 WT mice, which was largely suppressed in mice lacking Smad3, with a 50% reduction in CD4^+^IL-17A^+^ cells (Figure 7A-7C). Real-time PCR and western blot analysis further supported this notion that deletion of Smad3 markedly suppressed the expression of TGF-β1, IL-6, a transcriptional factor RORγt, and its signature cytokine IL-17A locally in the grafted heart tissues (Figure 8A and 8B).

It has been reported that TGF-β1 is a key inducer for the Treg development via a Smad3-dependent mechanism [9]. We thus investigated the Treg response in the grafted heart tissues in Smad3 KO mice. Two color immunofluorescence and flow cytometry analysis showed that deletion of Smad3 impaired the Treg response in the grafted heart tissues as demonstrated by a significant decrease in CD4^+^Foxp3^+^ cells (Figure 7D-7F), which was further confirmed by real-time PCR and western blot analysis with a marked inhibition of the Foxp3 transcriptional factor and IL-10 expression (Figure 8C and 8D).

Interestingly, consistent with the finding in the grafted heart, real-time PCR detected expression of Foxp3 and IL-10 mRNA in the spleen was suppressed in Smad3 KO recipients, which was associated with inhibition of CD4^+^Foxp3^+^ Treg population as demonstrated by ELISA and two-color flow cytometry analysis (Figure 6D-6F). In contrast, Th17 response in the spleen was increased in Smad3 KO mice when compared to Smad3 WT mice during cardiac allograft rejection (Figure 6D-6F).

## DISCUSSION

The present study demonstrated that mice lacking Smad3 developed less severe allograft rejection and improved the allograft survival rate in cardiac transplantation. This protective effect on acute cardiac allograft rejection was associated with a marked inhibition of cardiac inflammation and Th1 and Th17 immune response. However, we also found that mice null for Smad3 promoted Th2 immunity while impairing the Treg cell development. Results from this study suggested that Smad3 is an important regulator of T cell immunity and differentially regulates the T cell immune response during acute cardiac transplantation rejection.

It is well known that TGF-β plays a crucial role in T cell survival, proliferation and differentiation and is required for induction and maintenance of transplantation tolerance by induction of Treg cells [1, 4, 10-12]. Results from this study demonstrated that Smad3 was a key regulator of TGF-β signaling that diversely regulates the T cell immunity during cardiac allograft rejection. It is now clear that TGF-β/Smad3 signaling is essential for the induction of Foxp3 and the development of Tregs [13], which is critical in the induction and maintenance of transplantation tolerance. Smad3 binding to the enhancer of Foxp3 gene is a necessary step in Treg induction [13, 14]. The finding from this study showed that mice lacking Smad3 impaired the Treg cell development systemically, resulting in a loss of Treg response locally in grafted heart. This suggests a crucial role for Smad3 in TGF-β-induced immune tolerance during transplant rejection. However, consistent with a previous finding in skin transplantation rejection [15], deletion of Smad3 resulted in the improvement of the cardiac allograft survival despite of the loss of Treg cells. Although outcome from this study did not fully support the essential role for TGF-β/Smad3 signaling in the induction of transplantation tolerance, it does suggest the complexity of Smad3 in regulating allograft rejection, which was further discussed below.

In the present study, we found that deletion of Smad3 resulted in the suppression of the Th17-mediated allograft rejection as detected by a marked inhibition of the transcriptional factor RORγt and its signature cytokine IL-17A in the donor heart. In contrast, deletion of Smad3 promoted Th17 development with higher levels of Th17 response in the spleen of Smad3 KO recipients. This discrepant finding suggests that differential mechanisms may operate in the local versus systemic Th17 responses. It is well established that Th17-driven immune response plays a critical role in acute allograft rejection [16]. In the presence of IL-6, TGF-β1 induces the generation of IL-17-producing T cells [17, 18]. In the present study, we found that Smad3-deficency inhibited both TGF-β1 and IL-6 expression locally in the transplanted heart, thereby suppressing RORt-driven IL-17A expression and CD4^+^IL17A^+^ cell-mediated acute cardiac allograft rejection. This finding was consistent with a previous study that inhibition of Smad3 attenuates Th17-mediated skin lesions in a mouse model of psoriasis [19]. In contrast, higher level of Th17 response was found in the spleen of Smad3 KO recipients. This may be associated with the loss of Foxp3 because Foxp3 can inhibit Th17 cell differentiation by antagonizing RORγt [20]. Thus, it is highly possible that loss of Smad3 may suppress Th17-mediated acute allograft rejection locally by blocking its upstream signaling including inhibition of TGF-β1 and IL-6. However, deletion of Smad3 may promote the Th17 development systemically in the lymphoid tissues due to the loss of Foxp3-dependent inhibition of RORγt expression.

An interesting finding in this study was that loss of Smad3 from the recipients resulted in a shift of immune response from the Th1 to the Th2 in cardiac allografts, which is consistent with the alloskin rejection [15]. This shift from the Th1 to Th2 immune response locally in the grafted heart was associated with the impaired Th1 but enhanced Th2 differentiation systemically in the spleen tissue. Generally, Th1 cells are responsible for the induction and maintenance of the cellular immunity, while Th2 cells drive the humoral response [21]. In transplant rejection, Th1 cells promote allograft rejection characterized by increasing the Th1 reactivity with high levels of IL-2 and IFN-γ within the grafted tissues [22]. Th2 cytokines, particularly IL-4 and IL-10, are believed to inhibit the Th1 response in allograft rejection [23]. In the present study, the shift of allograft rejection from Th1 to Th2 response may be another mechanism whereby Smad3-deficiency improved allograft survival, although mechanisms remain largely unclear. It has been shown that Smad3 is essential for TGF-β1 signaling to inhibit the differentiation of Th1 and Th2 subsets [24, 25]. However, results from our study did not fully support this notion, suggesting Smad3-dependent and independent mechanisms in the regulation of the Th1/Th2 immunity. It is well established that differentiation of CD4^+^ T cells to the Th1 phenotype is regulated by T-bet, a Th1-specific transcription factor that initiates Th1 development while inhibiting Th2 cell differentiation [26, 27]. In contrast, GATA-3, a member of the GATA family of zinc finger proteins, plays a pivotal role in the development of the Th2 phenotype while inhibiting Th1 cells [28]. The interrelation between the T-bet and the GATA-3 determines the balance of Th1 and Th2 development [29]. T-bet has a binding ability to GATA-3, CBP/P300, and Sp1 to form a transcription factor complex [29]. The principal function of T-bet in developing Th1 cells is to negatively regulate GATA-3 [29, 30]. Thus, T-bet deficient CD4^+^ T cells are skewed toward Th2 differentiation by high endogenous GATA-3 level [29]. Whereas, GATA-3 shuts down Th1 and induces Th2 development through the repression of Stat 4 and augments its own expression by a positive feedback autoregulation mechanism [28, 30]. It is found that Smad3 is physically associated with GATA-3 and treatment of T cells with TGF-β promotes the formation of Smad3/GATA-3 nuclear complex and regulates IL-5 promoter activity and IL-10 production in a Smad3- and GATA-3-dependent manner [31]. This finding may explain the signaling mechanism by which TGF-β inhibits IL-4-induced GATA-3 expression on CD4^+^ T cells and suppresses IL-4-mediated Th2 differentiation [32]. Thus, deletion of Smad3 resulted in a loss of the inhibitory effect of TGF-β on GATA-3. Once GATA-3 is upregulated, expression of T-bet is suppressed, thereby promoting Th2 but suppressing Th1 mediated cardiac allograft rejection. Results obtained from this study also support the notion that the T-bet/GATA-3 ratio is important in determining the balance of Th1/Th2 immune response [33]. However, in this study, deletion of Smad3 inhibited IL-10 expression, which was inconsistent with previous study [15]. Since IL-10 is an important pleiotropic immunoregulatory cytokine secreted by different cell types, including macrophages, Th2 cells, and Treg cells et al [34], It is highly possible that inhibition of IL-10 expression in Smad3 KO mice during cardiac allograft rejection may be associated with inhibition of macrophage infiltration and importantly the loss of Treg response.

Inhibition of innate immunity may also account for the protective effect on cardiac allograft rejection in Smad3 KO mice. Recent studies showed that allograft rejection mounted by adaptive immune cells is shaped by the innate immunity [35]. Among them, macrophages are a key mediator [36]. Consistent with this finding, we also found that macrophage infiltration to the cardiac allograft in Smad3 KO recipients was less prominent. This may be associated with the impairment of Smad3 KO monocytes to the chemotaxis to TGF-β1 since Smad3 is a critical for TGF-β-induced MCP-1 expression [37, 38]. In addition, mice lacking Smad3 exhibited a marked inhibition of neutrophil infiltration and expression of proinflammatory cytokines IL-1β, TNF-α, and MCP-1. All these findings are consistent with many previous studies in which deletion of Smad3 protects against inflammatory response during ischemic and hypertensive cardiac remodeling [38, 39].

In summary, deletion of Smad3 from the recipients results in the improvement of cardiac allograft survival, which may be associated with inhibition of the Th1 and Th17 immune response. However, Smad3-deficiency also results in a loss of Treg cells and promotes Th2-dependent humoral cardiac allograft rejection. Results from this study suggest that Smad3 is a critical immune regulator in maintaining the T cell immunity during transplantation rejection.

## MATERIALS AND METHODS

### A mouse model of acute cardiac allograft rejection

Smad3 KO and wild-type (WT) mice (H-2^b^, both sexes, age 8 weeks, *n* = 6 /group), congenic to the C57BL/6 strain [7], and BALB/c mice (H-2^d^, both sexes, age 8 weeks), were used in this study. A mouse model of cardiac allograft rejection was induced by transplanting the donor hearts from BALB/c mice into the abdomen of Smad3 KO and WT mice [40]. Cardiac transplantation of donor hearts from BALB/c mice into the abdomen of BALB/c mice was used as control. All transplant experiments were performed using sex-matched animals. Manipulations were performed according to the Department of Health (Hong Kong) guidelines in Care and Use of Animals and with approval of the Animal Experimentation Ethics Committee at the Chinese University of Hong Kong. Graft viabilities were assessed by daily abdominal palpation. Undetectable heart impulses for two consecutive days were considered as rejection.

### ELISA

To test humoral immune response, serum immunoglobulin levels were measured by enzyme-linked immunosorbent assay (ELISA). Briefly, 96-well plates were coated with rabbit anti-mouse immunoglobulin G (IgG) (Sigma, St Louis, MO, USA) for overnight at 4°C and blocked with 2% BSA in PBS for 2 hours at room temperature. Then gradient dilutions of mouse serum in duplicate were added and incubated overnight. The bound anti-mouse IgG were detected with horseradish peroxadise (HRP) conjugated rabbit anti-mouse IgG (Dako, Glostrup, Denmark), IgG1 and IgG2a (Sigma) antibodies and the reaction optical density (OD) was determined at 405nm. Serum levels of IFN-γ, IL-4, IL-10 and IL-17A were tested using ELISA kit (Biolegend, CA, USA).

### Flow cytometry analysis

Graft heart and spleen tissues were digested by blenzyme 4 (Roche Inc, Indianapolis, IN, USA) into cell suspension. The white blood cells were then enriched by centrifugation through discontinuous Percoll (Pharmacia Fine Chemicals, Uppsala, Sweden) density gradients (40%, 60% and 80%). After being stimulated with Cell Stimulation Cocktail (plus protein transport inhibitors, eBioscience, San Diego, CA, USA) for 12 hours in CO_2_ incubator, the gathered cells were fixed for 30 minutes in IC Fixation Buffer (eBioscience) and permeablized by 1x Permeabilization Buffer (eBioscience) for 30 minutes. Then cells were stained with fluorescein isothiocyanate (FITC) -conjugated anti-mouse CD4, allophycocyanin (APC) -conjugated IFN-γ, phycoerythrin (PE) -conjugated IL-4, Foxp3-APC or IL-17A-PE (eBioscience) for overnight at 4°C. After being extensively washed, single cells were analyzed by FACSCaibur flowcytometer (BD Biosciences, San Jose, CA).

### Histology and immunohistochemistry

Grafts were fixed in formalin and embedded in paraffin for H&E stain and immunohistochemistry (IHC) as described previously [41]. Periodate-lysine-paraformaldehyde (PLP) fixed, OCT-embedded 5μm sections were stained with antibodies against mouse CD4 (Leinco, St. Louis, MO), IFN-γ (eBioscience), IL-4 (eBioscience), Foxp3 (eBioscience), IL-17A (Abcam, Cambridge, UK), F4/80 (Serotec, Oxford, UK), CD3 (Abcam), CD11c (eBioscience), Neutrophil Marker Antibody (NIMP-R14), IL-1β, tumor necrosis factor-α (TNF-α), and monocyte chemoattractant protein-1 (MCP-1) (Santa Cruz Biotechnology, Santa Cruz, CA, USA). In addition, immunoglobulin deposition was detected by immunofluorescence (IF) in the OCT-embedded snap-frozen sections (4μm) with antibodies including the PE-conjugated anti-mouse IgG (Rockland Immunochemicals, Gilbertsville, PA, USA), IgG1 (Santa Cruz), and IgG2a (South Biotech, Birmingham, AL). Positive cells and cytokines infiltration were quantified in at least 10 consecutive high power fields (×200, ×400) using Image-proplus 7.0 software and expressed as percent positive area or number of positive cells/cm^2^ as previously described [42].

### Real-time PCR

Total RNA from the heart and spleen were isolated using the RNeasy kit (Qiagen Inc, Valencia, CA, USA). mRNA expression of IL-1β, MCP-1, TNF-α, T-bet, IFN-γ, GATA3, IL-4, RORγt, IL-17A, TGF-β1, IL-6, Foxp3, IL-10 and glyceraldehyde-3-phosphate dehydrogenase (GAPDH) was detected by real-time polymerase chain reaction (PCR) with the Opticon 2 Real-Time PCR detector (Bio-Rad) using the primers as previously described [19, 42-45] and below: GATA3: forward 5′-TCTGGAGGAGGAACGCTAAT-3′, reverse 5′-TTCGGGTCTGGATGCCTTCTTT-3′; IL-4: forward 5′-TTCTCGAATGTACCAGGAGCCA-3′, reverse 5′-TCGTTGCTGTGAGGACGTTT-3′; IL-10: 5′-AAGGGTTACTTGGGTTGCCA-3′, reverse 5′-TGCTCCACTGCCTTGCTCTTAT-3′; RORγt: forward 5′-TGTCCTGGGCTACCCTACTG-3′, reverse 5′-GTGCAGGAGTAGGCCACATT-3′. The ratio of interested mRNA against GAPDH was calculated and expressed as the mean ± standard error of the mean (SEM).

### Western blot analysis

Protein from graft tissues was extracted with RIPA lysis buffer for western blot analysis as described previously [38]. Briefly, after blocking, membranes were incubated overnight at 4°C with primary antibodies against T-bet (Santa Cruz), GATA3 (Abcam), Foxp3, RORγt (Abcam), or GAPDH. After washing, membranes were incubated with LI-COR IRDye 800–labeled secondary antibodies (Rockland). The signal was detected with Odyssey Infrared Imaging System (Li-COR Biosciences, Lincoln, NE) and quantified against the internal loading control GAPDH with ImageJ version 1.48 (NIH, Bethesda, MD). The ratio of interested protein against GAPDH was calculated and expressed as the mean±SEM.

### Statistical analyses

Data obtained from this study are expressed as the mean±SEM. Statistical analyses were performed using t test and one-way analysis of variance (ANOVA), followed by Newman-Keuls' multiple comparison test using GraphPad Prism 5.0. The graft survival curves were calculated by the Kaplan–Meier method and the log-rank test was used for graft survival comparison. A *p* value < 0.05 was considered statistically significant.
